# The Army Bill and the Medical Corps

**Published:** 1899-03

**Authors:** 


### THE ARMY BILL AND THE MEDICAL CORPS.

PREVIOUS to the declaring of the Spanish-American war, the army of the United States was attended by a medical corps numbering about 190 surgeons. That was when peace reigned. When peace reigns, death's field lies fallow.

Then came the whirring of the wings of war. With war comes the fellow-fiends—death, disease and devastation.

The Surgeon-General was brought face to face with a grave responsibility. He had the health of a great and rapidly increasing army to care for. Under the provisions of a wise law he employed contract surgeons who were engaged to do regular army surgeon's work at so much per month without rank, without standing.

Each day saw the army increased by thousands; for war is the cradle of heroes and the tomb of cowards.

The Surgeon-General added to his staff of surgeons day by day as the army grew. American soldiers face the enemy with a song, but they cannot fight disease with bullets.

The war was fought and won and America became a nation with colonies. To govern a colony it must first be garrisoned. Government is power; power must have stability and support, else it becomes a mockery and the prey of fanatics. Stable government must be housed in barracks not in pest houses.

With the close of the war came the Hull bill increasing the army to 100,000 men. The department staffs were proportionately increased. To the medical corps were added 360 surgeons.

Then politics stepped in. Jealously, envy and inhumanity are foster-brothers of politics. They damn science and bless gold.

Provision was made in the amended bill to increase the army to 50,000, an increase of 100 per cent. To the medical corps was added 43 vacancies—an increase of 20 per cent. Fifty-three doctors to care for 25,000 soldiers, who will spend three-fourths of their service in the tropics. Congressmen are obedient children. They are begot of party and politics and they strangle their own flesh and blood at the behest of these unnatural parents.

Build bridges, plan conquests, expand, become the great, the greater, the greatest Republic—then bury the dead.

Victory comes not to disease-ridden armies.

Commanders who have led men into battle tell us how much more confident are their soldiers when they know the surgeons are ready for the work which is to come. They lack vim in proportion to the lack of surgeons. Congress cannot conquer territory by a two-thirds vote.

Fortunately for the men who carry rifles and wear the blue, the Surgeon-General has the right under certain circumstances to employ contract surgeons and he would be remiss in his duty to his God and his profession, if he permitted the aggregate strength of his corps at any time to fall below the original number as compared with the strength of the army.

The American soldier may fight his battles on a diet of canned roast beef; he might fight against an unequal and unmerciful strange foe, but he falls before disease.

No one can blame the Surgeon General for shortcomings when as executive head of the most important bureau of the army he is not permitted by a short-sighted, policy-infatuated congress to have what is necessary for the preservation of the health of the army—a sufficient number of doctors.

Send the soldier to the tropics and deprive him of sufficient medical attention and he will remain where he is sent; unless perhaps the government in a burst of anti-election generosity provides for the transportation of his body to the land of his birth.

